# Health-related quality of life and psychological distress in young adult survivors of childhood cancer and their association with treatment, education, and demographic factors

**DOI:** 10.1007/s11136-017-1716-0

**Published:** 2017-10-31

**Authors:** Janne F. Halvorsen, Anne Mari Sund, Lonnie Zeltzer, Marian Ådnanes, Heidi Jensberg, Terje A. Eikemo, Bendik Lund, Odin Hjemdal, Trude Reinfjell

**Affiliations:** 1 Department of Psychology, Norwegian University of Science and Technology (NTNU), 7491 Trondheim, Norway; 2 Department of Child and Adolescent Psychiatry, St. Olavs University Hospital, Trondheim, Norway; 3 Regional Centre for Child and Youth Mental Health and Child Welfare, Norwegian University of Science and Technology, Trondheim, Norway; 4 Department of Pediatrics, David Geffen School of Medicine at UCLA, Los Angeles, USA; 5 Department of Health Research, SINTEF Technology and Society, Trondheim, Norway; 6 Division of Health Economics and Financing, Norwegian Directorate of Health, Trondheim, Norway; 7 Department of Sociology and Political Science, Norwegian University of Science and Technology, Trondheim, Norway; 8 Department of Pediatrics, St. Olavs University Hospital, Trondheim, Norway; 9 Department of Laboratory Medicine, Norwegian University of Science and Technology, Trondheim, Norway

**Keywords:** Childhood cancer, Survivors, HRQOL, Psychological distress, Young adults

## Abstract

**Purpose:**

This study investigated health-related quality of life (HRQOL) and psychological distress among young adult (YA) survivors of childhood cancer and the association of these measures with treatment, education, and demographic factors ≥ 5 years post diagnosis.

**Methods:**

Participants included cancer survivors (*n* = 91) recruited through the Cancer Registry of Norway (CRN) and healthy controls (*n* = 223) recruited from a student population. All participants completed self-report questionnaires, and the Pediatric Quality of Life Inventory (PedsQL™) 4.0 and the Hopkins Symptom Checklist-10 (HSCL-10) as a measure of HRQOL and distress, respectively.

**Results:**

Survivors reported HRQOL at the same level as controls, except for poorer physical functioning. Survivors in general, and female survivors specifically, had higher odds than controls of reporting symptoms of distress above cut-off, but survivors did not have higher mean levels of distress compared to controls. Survivors reporting distress levels above the cut-off had significantly poorer HRQOL regarding physical functioning and lower total PedsQL scores than controls scoring above the cut-off. Age (for HRQOL only), female gender, low educational level, and perceived low economic status significantly predicted HRQOL and distress. Education interacted with the effect of cranial radiation in predicting HRQOL.

**Conclusions:**

Survivors reported similar mean levels of HRQOL and distress as controls, except for physical functioning. For cancer survivors, demographic variables predicted HRQOL and distress. Some groups of survivors require closer follow-up, and more attention should be paid to factors associated with poor HRQOL and psychological distress in survivors, including female gender, lower education level, and lower income. Survivors treated with cranial radiation may be particular vulnerable in combination with low education regarding HRQOL.

## Introduction

Improvements in survival rates for childhood cancers have contributed to an increased focus on late effects of treatment among long-term survivors (≥ 5 years post diagnosis) [1]. Although serious problems may occur during cancer therapy or soon after, the majority of problems do not become clinically apparent until years after the cancer has been cured [2]. This highlights the need for young adult (YA) survivors of childhood cancers to be evaluated for long-term effects when transitioning into adult-centred health care [3]. Nevertheless, the majority of YA survivors do not receive risk-based survivorship care to assess physical and psychosocial late effects [4].

Long-term follow-up studies of childhood cancer survivors suggest that although most adult survivors are psychologically healthy, certain subgroups are at risk for significant psychological distress (distress), including symptoms of depression, anxiety, and somatization [5–7]. Zeltzer et al. [6] found that risk factors for distress and poor HRQOL were female gender, lower educational attainment, unmarried status, lower household income, unemployment, lack of medical insurance, having a major medical condition, and treatment with cranial radiation. Compared to the paediatric and older adult populations, YA survivors may have different psychosocial concerns, typically related to establishing identity, and separating from parents and issues regarding career or employment decisions and higher education. In addition, health-related concerns about the future, and whether they will be able to establish their own family, as underlined by Zebrack et al. [8], may contribute to persistent distress for YA survivors. This increases the need for knowledge on how disease and treatment affect later the psychological functioning as well as health-related quality of life (HRQOL). HRQOL is a multidimensional construct covering physical, emotional, mental, social, and behavioural components of well-being and functioning as perceived by patients [9, 10].

Negative psychosocial outcomes and poor HRQOL are associated with female gender [6, 7, 11–14], and worse physical and mental adaptation are associated with older age at the time of assessment [12–14]. Individuals diagnosed between the age of 15 and 20 are nearly twice as likely to use antidepressant medication as compared to individuals diagnosed before the age of 5 [15], which may suggest an increased risk of mental health conditions among YA survivors [16, 17]. On the other hand, YA survivors may show considerable resilience and are more likely to report better social functioning and HRQOL compared with controls, despite diminished educational attainment and perceived social support [18]. Tremolada et al. [18] found that survivors demonstrated an even better perception of their lives than controls, and, according to the authors, this finding illustrates a profile of relatively good psychological health and resilience in paediatric cancer survivors. The results indicate that YAs can have normal development in health perceptions compared to their peers [18]. Young people who are able to accept and cope with cancer may gain greater appreciation of life as a result of their illness experience, may mature faster than their peers, and can become better equipped emotionally to handle the everyday challenges of life [8, 19].

This study aims to examine late effects in YA survivors of childhood cancer on self-reported HRQOL and distress and the association of those effects with treatment modalities, education, and demographic factors. First, we compare survivors and controls in levels of HRQOL and distress, differences in symptoms of distress (scores above cut-off), as well as gender differences in these constructs. Second, we investigate whether cranial radiation treatment, education, and demographic factors are associated with HRQOL and distress for this population of cancer survivors. It is hypothesized that no significant differences will be found between survivors and controls on HRQOL but survivors will report poorer physical health and higher levels of distress, including being more likely to score above the cut-off for distress. Female survivors are expected to have lower HRQOL and higher distress compared to female controls. Age, gender, lower educational attainment, lower household income, and treatment with cranial radiation are expected to be significant predictors of HRQOL and distress. In addition, to investigate moderation effects, it is hypothesized that gender will interact with education and treatment in predicting HRQOL and distress, where female survivors report poorer HRQOL and distress. Moreover, it is hypothesized that treatment with cranial radiation in interaction with education will predict poorer HRQOL and distress. To the best of the authors’ knowledge, this study contributes to the field of research by being the first to investigate these questions in a Norwegian YA population of childhood cancer survivors.

## Methods

### Study design and setting

The participating sample was derived from a previous study where participants were recruited through the Cancer Registry of Norway (CRN) by the research institution the Foundation for Scientific and Industrial Research at the Norwegian Institute of Technology (SINTEF). The birth cohorts were from 1980 to 1992. Age at survey was 18 years and above. All cancer diagnoses included in statistics from *Cancer in Norway* [20] were included. Of 536 survivors invited, 230 agreed to participate (42.9% response rate). In the present study, we included survivors based on the following main inclusion criteria: a follow-up time from diagnosis ≥ 5 years, and diagnosed ≤ 21 years of age and whose age at assessment was ≤ 29. Participant survivors included 56 females (61.54%) and 35 males (38.46%), with a mean age of 24.71 ± 2.77 years (range 20–29) (Table 1). Survivors were an average of 9.21 years (*SD* = 3.80) from diagnosis, with a mean age at diagnosis of 15.50 (*SD* = 3.83) years, diagnosed during 1991–2007 (Table 2). Controls were students recruited through lectures at the Norwegian University of Science and Technology (NTNU), and consisted of first through third year bachelor and clinical program psychology students, and fourth grade master degree teacher students, excluded by earlier or ongoing illness. The control group data were collected in January 2016. After matching with the survivor cohort on age (≤ 29 at assessments), the control group included 223 participants, 145 females (65.0%) and 78 males (35.0%), with a mean age 24.91 ± 2.05 years (range 20–32). There were no significant differences in gender, age, or perceived economic situation between survivors and controls.

**Table 1:** Demographic characteristics and differences between survivors and controls

Characteristic	Survivors (*n* = 91)		Controls (*n* = 223)		*t* or *χ* ^*2*^	*p*
	Frequency	%	Frequency	%		
Gender						
Female	56		145	65.0	ns	
Male	35		78	35.0		
Age mean (*SD*)	24.71 (2.77)		24.91 (2.05)		ns	
Educational level						
Not completed compulsory education	0	0.0	0	0.0	*t*(123) = − 5.26	0.000
Completed compulsory education	8	8.8	0	0.0		
Upper secondary school (11–13)	29	31.9	18	8.1		
College/university < 4 years	28	30.8	101	45.5		
College/university > 4 years	25	27.5	102	45.9		
Other	1	1.1	1	0.5		
Main occupation						
Working full time	37	40.7	0	0.0	*χ* ^*2*^ (5) = 143.19	0.000
Working part time	8	8.8	24	10.8		
Student	34	37.4	198	89.2		
Sick leave	0	0.0	0	0.0		
Social security benefit	11	12.1	0	0.0		
Other	1	1.1	0	0.0		
Perceived economic situation						
Very much above average	2	2.2	1	0.5	ns	
Above average	14	15.7	24	10.9		
Average	41	46.1	102	46.2		
Under average	27	30.3	72	32.6		
Very much below average	5	5.6	22	10.0		

**Table 2:** Cancer characteristics

	Frequency	%
Disease characteristic		
Leukaemia	13	14.3
Brain tumour	12	13.2
Testicular cancer	7	7.7
Ovarian cancer	2	2.2
Skin cancer/melanomas	2	2.2
Lymphoma	35	38.5
Other	23	25.3
Treatment		
Surgery or surgical procedure	56	61.5
Chemotherapy	72	79.1
Radiation	44	48.4
Hormonal treatment	11	12.1
Other treatment prescribed by doctor	5	5.5
Other treatments	10	10.1
Years since diagnosis		
5	15	16.5
6–7	25	27.5
8–9	13	14.3
Over 10	38	41.8
Mean years since diagnosis (*SD*)	9.21 (3.80)	
Age at diagnosis		
Mean (SD)	15.50 (3.83)	
0–10	8	
11–15	33	
16–21	50	

### Procedures

The survivor group data were collected during autumn 2012, with seven hospitals in Norway participating. Patient records were sent from the hospitals to CRN, who sent questionnaires to respondents. Respondents were informed that the CRN was used to identify them, and were instructed to return their questionnaires to SINTEF. Data were anonymized prior to statistical analysis. The study was approved by the Regional Committee for Medical and Health Research Ethics (2011/2647 and 2015/2218).

### Measures

Questionnaires and selected items used in the present study were based on earlier individual interviews with cancer survivors conducted in 2009. Seven cancer survivors, mean age 23.4 (range 18–25) years, all from the youth group of the Norwegian Cancer Society participated in the interview conducted by SINTEF.

Demographic questions included items regarding gender, age, living arrangements, economic situation (a lower value indicates better perceived situation), education, main occupation. For controls, one question regarding severe illness was added. Treatment modalities were self-reported by survivors, who indicated which of seven different treatments they had received.

A young adult version of the Pediatric Quality of Life inventory (PedsQL™) 4.0, originally developed by Varni et al. [21], was used to measure psychosocial health of AY cancer survivors. Structure and scales are described elsewhere [22]. The young adult version (18–25 years) was translated and linguistically validated in 2011. The validation of the adolescent version, practically identical to the young adult version, showed good psychometric properties [23]. PedsQL items were reverse-scored and linearly transformed on a scale ranging from 0 to 100, where higher scores indicate better HRQOL. Scale scores were computed as the sum of items divided by the number of items answered (this accounts for missing data) [22]. Cronbach’s alphas for scale internal consistency were between 0.89 and 0.92 for survivors and 0.82 and 0.90 for controls on subscales and main scale PedsQL; these findings are considered satisfactory [23].

The Hopkins Symptom Checklist-10 (HSCL-10) covers depressive and anxiety symptoms with five items each. Each item is rated on a scale from 1 (not at all) to 4 (extremely) [24]. Average item score was calculated by dividing the total score by the number of items answered. Records with three or more missing items were excluded, resulting in exclusion of one (1.10%) of total participants. The cut-off point for distress was set to 1.85 as recommended by Strand et al. [25]. A higher value on HSCL indicates higher level of reported distress. On HSCL, Cronbach’s *α* = 0.91 for cancer survivors and *α* = 0.88 for controls were ≥ than reported by Strand et al. [25].

### Statistical analyses

The Statistical Package for the Social Sciences (SPSS, version 23) was used to conduct statistical analyses. Two-sided *p* values < .05 were considered statistically significant. Cohen’s *d* and odds ratios were used to assess effect size. Regarding Cohen’s *d*, where homoscedasticity could not be assumed, the *s* (sample std. deviation) of the control group was used to calculate effect size, as recommended by Field [26]. Descriptive analyses were conducted. Independent samples *t* test and Pearson’s Chi-square test in crosstabs were used to estimate sociodemographic differences between survivors and controls. Cronbach’s alpha (α) was used to assess internal consistency of the questionnaires. Independent samples *t* tests, Pearson’s Chi square, ANOVA, and hierarchical stepwise regression analysis was used to investigate the hypotheses.

## Results

There were significant differences (*p* < .05) between survivors and controls in educational level and main occupation (Table 1). A majority of survivors were diagnosed with lymphomas (Table 2).

Comparing survivor and control means on PedsQL and HSCL revealed no significant differences on total scale PedsQL *t*(120) = − 1.47, *p* = .144, or HSCL *t*(133) = 1.45, *p* = .150. On physical functioning, mean score for survivors was significantly lower (less physical functioning) *t*(120) = − 2.13, *p* = .035, and represents a small effect size *d* = − 0.39 (Table 2).

Female survivors scored significantly lower than female controls on PedsQL physical functioning/health *t*(71) = − 2.32, *p* = .024. Using Pearson’s Chi square, there was a significant association between being in the female survivor cohort and scoring above the cut-off for distress on HSCL, χ^2^(1) = 11.52, *p* < .001. Of the female cancer survivors (*n* = 55), 47. 27% (*n* = 26) scored above the cut-off for distress versus 22.76% (*n* = 33) of female controls (*n* = 145). The odds of scoring above the cut-off for distress were 3.04 times higher for female survivors than for female controls, equal to *d* = 0.61, considered a medium effect size. For male survivors and controls, an independent samples t test resulted in no significant differences between means on PedsQL or HSCL. A Pearson’s Chi-square test did not reveal any significant association between type of group and scores above or below cut-offs for distress χ^2^(1) = 0.03, *p* = 1.00. Among men, 14.29% (*n* = 5) of cancer survivors (*n* = 35*)*, and 15.58% (*n* = 12) of controls (*n* = 77) scored above the cut-off.

As indicated by Pearson’s Chi square, there was a significant association between belonging to the survivor group and scores above the cut-off for distress on HSCL χ^2^(1) = 6.98, *p* = .009. The odds of scoring above cut-off were 2.07 times higher for survivors than for controls, equivalent to *d* = 0.40, a small effect size. In all, 34.44% (*n* = 31) of survivors (*n* = 90) reported symptoms at a level consistent with distress (scores above cut-off), while 20.27% (*n* = 45) of controls (*n* = 222) did the same. For the respondents scoring above the cut-off on HSCL, there were significant differences between survivors and controls on PedsQL: Physical functioning *t*(74) = − 2.43, *p* = .017 and total scale *t*(73) = − 2.22, *p* = .030 (Table 3). Effect sizes can be considered small or medium for PedsQL scales.

**Table 3:** Means and *p* values for PedsQL and HSCL from independent samples *t* test and Pearson’s Chi square

Scale	Survivors vs. controls						Female					Above cut-off				
		Survivors	Controls	*t*	*p*	*d*	Survivors	Controls	*t*	*p*	*d*	Survivors	Controls	*t*	*p*	*d*
Physical health	*N*	91	223				56	145				31	45			
	*M (SD)*	78.28 (22.68)	83.74 (14.18)	− 2.13	.035*	− 0.39	74.04 (24.10)	82.01 (14.68)	− 2.32	.024*	− 0.54	61.68 (22.06)	71.42 (16.43)	− 2.43	.017*	− 0.59
Total PedsQL	*N*	90	223				55	145				30	45			
	*M (SD)*	77.41 (17.33)	80.32 (11.20)	− 1.47	.144	− 0.26	73.91 (17.28)	78.95 (11.47)	− 2.00	.049	− 0.44	61.81 (13.30)	68.38 (12.05)	− 2.22	.030*	− 0.55
Psychosocial health summary	*N*	90	223				55	145				30	45			
	*M (SD)*	76.66 (17.00)	78.50 (11.32)	− 0.94	.347	− 0.16	73.40 (16.60)	77.32 (11.48)	− 1.61	.111	− 0.34	61.28 (11.85)	66.22 (11.35)	− 1.81	.074	− 0.44
HSCL	*N*	90	222				55	145				31	45			
	*M (SD)*	1.64 (0.64)	1.53 (0.49)	1.45	.150	0.22	1.75 (0.65)	1.58 (0.52)	1.80	.077	0.33	2.39 (0.48)	2.29 (0.46)	0.952	.344	0.20

*p < .05

Before running the hierarchical stepwise regression analyses, assumptions of collinearity were investigated. For PedsQL and predictors, no correlations had an absolute value above *r* = .512. For HSCL and predictors no correlations had an absolute value above *r* = .476. The variance inflation factor (VIF) was below 10 and tolerance statistic above 0.2 for all values, indicating no problems associated with collinearity in the data [26]. The following predictor variables: gender, age, education, economic situation significantly predicted PedsQL total scale and HSCL scale (Table 4). The specified interactions were investigated by adding one interaction term to the model at a time using mean-centred values. There were no effects of gender in combination with education or radiation treatment in predicting HRQOL and distress outcome. The only significant interaction was between cranial radiation treatment and education in predicting HRQOL (Table 4). Note that no main effect of cranial radiation was found, but in combination with low education, cranial radiation predicted poorer HRQOL.

**Table 4:** Hierarchical stepwise regression analysis predicting (a) PedsQL total scale (*n* = 88)^a^, (b) HSCL (*n* = 88)^b^

(a)						(b)				
Predictor	U.std. coefficients		Std. coefficients	*t*	*p*	U.std. coefficients		Std. coefficients	*t*	*p*
	B (95% CI)	Std. error	Beta			B (95% CI)	Std. error	Beta		
Gender	0.33 (0.15, − .51)	0.09	0.30	3.67	0.000**	− 0.29 (− .49, − 0.09)	0.10	− 0.26	− 2.89	0.005**
Age	− 0.20 (− 36, − 0.04)	0.08	− 0.21	− 2.47	0.016*	0.15 (− .03, 0.33)	0.09	0.16	1.71	0.091
Education	0.49 (0.33, − 0.65)	0.08	0.52	6.09	0.000**	− 0.46 (− .63, − 0.29)	0.09	− 0.50	− 5.32	0.000**
Perceived economic situation	− 31 (− .50, − 0.12)	0.10	− 0.27	− 3.29	0.001**	0.27 (0.05, 0.48)	0.11	0.22	2.46	0.016*
Radiation	− 0.02 (− .22, − .18)	0.10	− 0.02	− 0.173	0.863	− 0.06 (−.28, 0.16)	0.11	− 0.05	− 0.55	0.585
Education × radiation	0.18 (0.03, − .33)	0.08	0.20	2.31	0.023*	− 0.08 (− .241, − 0.090)	0.08	− 0.09	− 0.91	0.366

*p < .05, **p < .01

^a^Dependent variable: PedsQL total scale: Step 1: *R* ^*2*^ *cha* = 0.06, *p* = .018; Step 2: *R* ^*2*^ *cha* = 0.00, *p* = .832; Step 3: *R* ^*2*^ *cha* = 0.30, *p* < .000; Step 4: *R* ^*2*^ *cha* = 0.08, *p* = .001; Step 5: *R* ^*2*^ *cha* = 0.01, *p* = .274; Step 6: *R* ^*2*^ *cha* = 0.03, *p* = .023

^b^Dependent Variable: HSCL: Step 1: *R* ^*2*^ *cha* = 0.05, *p* = .039; Step 2: *R* ^*2*^ *cha* = 0.00, *p* = .940; Step 3: *R* ^*2*^ *cha* = 0.25, *p* < .000; Step 4: *R* ^*2*^ *cha* = 0.05, *p* = .010; Step 5: *R* ^*2*^ *cha* = 0.00, *p* = .829; Step 6: *R* ^*2*^ *cha* = 0.01, *p* = .366

## Discussion

We report on late effects in YA survivors of childhood cancer on self-reported HRQOL and distress and their association with treatment modalities, education, and demographic factors. Overall, the majority of survivors report HRQOL and distress at a level consistent with controls, but report poorer physical functioning on average 9.21 years post diagnosis. Similar levels in HRQOL and distress may seem counterintuitive, but might be explained by adaptive repression [27], response shift [28], or positive growth [29]. Results thus support earlier research regarding positive growth and resilience [29, 30], with the data from this study indicating that the same is true for Norwegian YA survivors of childhood cancer.

Odds of scoring above the cut-off were 2.07 times higher for cancer survivors than for controls. This may seem counterintuitive given the non-significant difference between the two groups’ means for distress. However, the greater variance in the survivor group suggests that only a subgroup of survivors report large amounts of distress. Additionally, unlike controls in prior studies, the control population in this study was recruited from a college sample that also reported high levels of distress (in all, 34.44% of survivors and 20.27% of controls). Controls in our study demonstrated higher levels of distress than the controls in an earlier study, where only 11.4% scored above the cut-off [25]. Furthermore, a higher cut-off was used in the current study than in a prior study [31], a finding suggesting that both survivors and controls comprised stressed populations in the current study. For participants scoring above the cut-off for distress, there were significant differences between survivors and controls on physical and total HRQOL, despite no significant difference on scores of distress. This finding suggests that clinical symptoms of distress may have a greater impact on HRQOL for survivors than for controls. The fact that survivors might be more vulnerable to the impact of high distress on HRQOL complements earlier research findings [5, 16, 32] and might imply greater impairment in daily living or mirror different kinds of stress in the two groups. This relationship between high distress and poor HRQOL underscores the importance of measuring and assessing these constructs during long-term follow-up.

Consistent with our hypotheses and earlier research [7, 14, 15], female survivors score lower on physical HRQOL than do female controls and controls in general. This may result from greater vulnerability to treatment-related toxicities among women [32] or it may reflect similar trends in the general population [32]. Female survivors had 3.04 times higher odds of scoring above the cut-off than female controls, findings that suggest female survivors are more vulnerable to these outcomes than are women in general.

Regression analyses support the following factors associated with poor HRQOL and high levels of distress among YA survivors: being female, older age at time of assessment (for HRQOL only), having less education, and reporting poor economic status. No effect of gender in combination with education or cranial irradiation was found in predicting HRQOL and distress. However, while no main effect of cranial radiation treatment was found, in combination with low education, cranial radiation predicted poor HRQOL. This demonstrates that survivors with low educational status and who have undergone radiation treatment may be more vulnerable regarding HRQOL outcome, suggesting which groups are suitable for interventions. Educational level was the strongest predictor of HRQOL and distress. Findings from regression analyses combined with negative findings regarding differences in HRQOL and distress related to treatment indicate that important non-treatment-related factors impact quality of life in the survivor group. Consistent with other research, our findings propose gender, educational level, and perceived economic situation as additional risk factors [6, 7, 12, 13, 33]. Recent findings indicate that Norwegian cancer survivors (aged 22–42) diagnosed before the age of 25 have an increased risk of being economically dependent and unemployed [34], a factor that could help explain the finding of economic status as a risk factor in our study. Predicting levels of HRQOL and distress from demographic variables is by and large in accordance with the study by Zeltzer et al. [7].

Study findings indicate that subgroups of YA survivors are at risk for significant distress and poor HRQOL years after diagnosis. These findings emphasize the need to ensure long-term follow-up of survivors, provide resources to implement this type of assessment and care, and enhance knowledge among health care personnel, school personnel, and the education system in general about these long-term effects.

The present findings have several limitations. The low response rate of the study limits the generalizability of the findings, and, unfortunately, information on non-participants was not available. While a relatively low response rate creates uncertainties around our estimates, our findings support prior research on YA survival rates [35]. One might hypothesize only the healthiest and most highly educated survivors participated, while survivors with diminished HRQOL were less likely to participate. Further, a cross-sectional design will prevent the estimation of causal relationship among variables. Longitudinal studies are required to broaden knowledge about psychological distress and HRQOL of YA survivors over time. Small effect size limits conclusions, and differences between groups in some demographic characteristics may have impacted findings. Comparing means of PedsQL and HSCL-10 to earlier studies using these measures [23, 25, 36], the lower HRQOL control group scores in our study support data from other student populations [22]. The data collection for the survivor group and controls took place during the same period of the year, in the winter, for both groups. There are 4 years between the data collection for the survivor and control groups, but it is important to be aware that no major events of particular importance in the Norwegian society occurred during this period, and there were no major changes or reforms in the Norwegian health care or university systems that would indicate that the terms are different for the survivor and control groups. Further, and perhaps even more importantly, Norway is one of the last countries in Europe to offer free access to tertiary education. The principle of free education is a prerequisite for Norwegian higher education and the most important instrument for ensuring equal rights to education. While free education is essential for democracy and community development, it also promotes social mobility. Norwegian university students, therefore, are a less selective group in terms of wealth and health compared to other countries (one in four attends university in Norway). Still, we acknowledge that this is a limitation, even in a Norwegian sample (there is also some degree of social reproduction in Norway).

The fact that nobody from the comparison group was working full time, while 40% of the survivor group was, and its implication for HRQOL and distress is an important detail. The reason that 40% of the survivors were working full time could be related to their education level, as the results show (see Table 1) that the level of education is lower for the survivor group compared to the control group. However, since survivors have not been in the educational system as long as the controls, it may be natural and even positive that as many as 40% are working full time; at the same time, it also may indicate that members of the survivor group have not been able to study or complete higher education, and this should be investigated in further studies. However, being able to work can be a positive factor and may influence the similarity in HRQOL reported by the survivor group and the control group (except for physical functioning). Interestingly, this does not result in differences between the groups in how they subjectively experience their economic status. However, low educational level and perceived low economic status significantly predicted HRQOL and distress for the survivors group, and should be a focus in further studies. The PedsQL measure has not yet been validated for this age group in Norway, and thus future research should carry out validation studies in Norwegian populations in the age group studied in this investigation. Strengths of our study include having a large survivor cohort representing patients from several Norwegian hospitals followed for a significant amount of time after diagnosis (9.21 years, *SD* = 3.83).

## Conclusion

Norwegian survivors report poorer physical HRQOL, but not poorer total or psychosocial HRQOL, than controls. Nor do they report higher distress than controls. This finding could represent an effect of resilience, positive growth, or lowered self-expectations. Despite showing no significant differences in levels of distress, survivors have higher odds of scoring above the cut-off for distress, and the impact of high levels of distress on HRQOL seems to be stronger for survivors than for controls. In line with earlier research, we found females to be an at-risk group. Female survivors scored significantly lower on physical HRQOL and had higher odds of being distressed than did female controls. Age (only for HRQOL), gender, education, and perceived economic situation significantly predict HRQOL and distress. In addition, survivors treated with cranial radiation may be particular vulnerable in combination with low education regarding HRQOL, and should be investigated in further research with longitudinal studies. Insights from this study can be used to identify at-risk survivors so that appropriate care can be provided.
